# *MicroRNA*s: New Therapeutic Targets for Familial Hypercholesterolemia?

**DOI:** 10.1007/s12016-017-8611-x

**Published:** 2017-05-22

**Authors:** Amir Abbas Momtazi, Maciej Banach, Matteo Pirro, Evan A. Stein, Amirhossein Sahebkar

**Affiliations:** 1 Nanotechnology Research Center, Department of Medical Biotechnology, Faculty of Medicine, Mashhad University of Medical Sciences, Mashhad, Iran; 2 Department of Hypertension, WAM University Hospital in Lodz, Medical University of Lodz (MUL), Zeromskiego 113, 90-549 Lodz, Poland; 3 Polish Mother’s Memorial Hospital Research Institute (PMMHRI), Lodz, Poland; 4 Cardiovascular Research Centre, University of Zielona Gora, Zielona Gora, Poland; 5 Unit of Internal Medicine, Angiology and Arteriosclerosis Diseases, Department of Medicine, University of Perugia, Perugia, Italy; 6 Metabolic and Atherosclerosis Research Center, Cincinnati, OH USA; 7 Biotechnology Research Center, Mashhad University of Medical Sciences, Mashhad, 9177948564 Iran; 8 School of Medicine, University of Western Australia, Perth, Australia; 9 Department of Medical Biotechnology, School of Medicine, Mashhad University of Medical Sciences, P.O. Box: 91779-48564, Mashhad, Iran

**Keywords:** Apolipoprotein B, Low-density lipoprotein cholesterol, LDL receptor, LDLRAP1, microRNA, PCSK9

## Abstract

Familial hypercholesterolemia (FH) is the most common inherited form of dyslipidemia and a major cause of premature cardiovascular disease. Management of FH mainly relies on the efficiency of treatments that reduce plasma low-density lipoprotein (LDL) cholesterol (LDL-C) concentrations. MicroRNAs (miRs) have been suggested as emerging regulators of plasma LDL-C concentrations. Notably, there is evidence showing that miRs can regulate the post-transcriptional expression of genes involved in the pathogenesis of FH, including *LDLR*, *APOB*, *PCSK9*, and *LDLRAP1*. In addition, many miRs are located in genomic loci associated with abnormal levels of circulating lipids and lipoproteins in human plasma. The strong regulatory effects of miRs on the expression of FH-associated genes support of the notion that manipulation of miRs might serve as a potential novel therapeutic approach. The present review describes miRs-targeting FH-associated genes that could be used as potential therapeutic targets in patients with FH or other severe dyslipidemias.

## Necessity of New Therapeutic Options for FH Patients

Familial hypercholesterolemia (FH) is a frequent, severe, and mostly autosomal dominant genetic disorder associated with elevated plasma low-density lipoprotein cholesterol (LDL-C) levels that predisposes patients to premature cardiovascular disease (CVD), especially if remain undiagnosed or inadequately treated [1–3].

The disorder most commonly results from loss-of-function mutations in the *LDLR* gene encoding LDL receptor protein, and genes encoding for proteins that interact with the receptor, including *apolipoprotein B (APOB)*, *proprotein convertase subtilisin/kexin type 9* (*PCSK9)*, or *LDLR adaptor protein 1* (*LDLRAP1)* [4, 5]. These pathogenic mutations cause deficient clearance of circulating LDL particles via hepatic LDLR leading to increased plasma LDL-C levels from birth and deposition in the arterial wall, thus accelerating atherosclerosis and the risk of premature CVD [6, 7].

Statins, ezetimibe, bile acid sequestrants, and more recently PCSK9 inhibitors are the main therapeutic drugs for the treatment of heterozygous FH (HeFH); all of which work solely or predominantly via increased LDLR activity and LDL-C clearance. Additional LDL-C-lowering can be achieved in homozygous FH (HoFH) patients by decreasing production of LDL-C, or its precursors, with either the microsomal triglyceride transfer protein (MTP) inhibitor, lomitapide, or the antisense oligonucleotide which reduces apoB synthesis, mipomersen. LDL-C reduction can also be achieved by regular LDL apheresis which mechanically removes LDL. Statins and ezetimibe are the most commonly used cholesterol-lowering drugs and are now generically available at low cost in most countries [8–10]. Nevertheless, despite the 50 to 60% LDL-C reduction achievable by these two generally well-tolerated agents, a large number of FH patients still do not reach the recommended LDL-C goals due to their very high baseline LDL-C levels [2, 3]. In HeFH patients who already have evidence of CVD, less than 10 to 20% achieve a LDL-C level below 70 mg/dL on conventional drug therapy [11]. In HoFH patients, where the typical phenotype has untreated LDL-C > 500 mg/dL and at only minimal to moderate residual LDLR activity, even high doses of atorvastatin or rosuvastatin reduce mean LDL-C by only 22–25% [12, 13], and ezetimibe achieves an additional 20% reduction [14], thus few, if any, HoFH patients can reach anywhere near optimal LDL-C levels.

Therefore, alternative therapeutics either alone or in combination with current approved therapies can be considered to reduce residual disease burden in patients with FH [15]. In this context, modulation of microRNAs (miRs)-dependent gene expression represents a promising approach.

## RNA-Based Therapeutic Approaches

In the past decade, RNA-based approaches have shown potential as novel therapies for human disorders. These therapeutic approaches are indebted to the advances in knowledge of the RNA field. As revealed by ENCODE (Encyclopedia of DNA Elements) project, a multi-center study aiming to find a collection of functional elements in the human genome via sequencing RNA from various sources, more than 90% of the human genome contains non-coding RNAs that can affect other coding sequences of the genome [16]. Several new classes of non-coding RNAs associated with the most disparate and critical functions have been identified [17]. Among these, small interfering RNAs (siRNAs) and miRs have attracted considerable interest for drug discovery and development because of their important role in gene regulation [18].

### RNA Interference Pathway

miRs and siRNAs are endogenously transcribed non-coding short hairpin RNAs that inhibit gene expression through RNA interference (RNAi) pathways [19–21]. RNAi is a highly conserved cellular mechanism present in the most eukaryotic cells that interferes with post-transcription steps and consequently silences the expression of homologous genes [21, 22]. Either double-stranded miRs or siRNAs, composed of a passenger strand (sense strand) and a guide strand (antisense strand), can interact with and activate the RNA-induced silencing complex (RISC). The passenger strand is cleaved by the endonuclease argonaute 2 (AGO2) of the RISC, while the guide strand remains associated with the RISC. Subsequently, the guide strand directs the active RISC to target mRNA that is cleaved by AGO2 component [22, 23]. miRs inhibit the expression of genes by hybridizing guide strand to partially complementary binding sites typically localized in the 3′ untranslated regions (3′ UTR) of target mRNAs, while siRNA guide strand only binds to mRNA that is fully complementary to it, causing specific gene silencing [24, 25]. In rare cases, mRNAs contain highly complementary miRNA-binding sites and therefore miRs guide the sequence-specific cleavage of the mRNA in a process similar to that mediated by siRNAs [25]. Mechanistically, efficient inhibition is either supplied by interfering with translation or by predisposing mRNAs to degradation that is initiated by deadenylation and decapping of the mRNAs [24].

### Therapeutic Potential of RNAi-Based Therapeutics

The therapeutic potential of miRs and siRNAs has been shown in the treatment of many different diseases such as cancers [26–29], infections [29–32], and cardiovascular diseases [33, 34]. The siRNA-based therapeutic approaches involve the delivery of a synthetic siRNA into the target cells aimed at suppressing the expression of a specific mRNA, and provide a gene silencing effect [35]. The siRNA-based cholesterol-lowering agent inclisiran (formerly ALN-PCS) has recently successfully completed a phase II trial in 497 patients with a baseline LDL-C of ~130 mg/dL [36]. Patients receiving a single subcutaneous injection of 300 mg of inclisiran achieved mean LDL-C reductions of 51 and 45% at Days 60 and 90, respectively (*p* < 0.0001 compared to placebo). Inclisiran was generally well tolerated with treatment emergent adverse events of 54% both in patients randomized to placebo or inclisiran, and with no differences between inclisiran doses. Injection site reactions (ISRs) with inclisiran were seen in 3.2% of patients, and were mild or moderate, and mostly transient. Inclisiran thus appears to provide a durable reduction in PCSK9 and LDL-C levels with small and infrequent subcutaneous injections [36].

In contrast to the siRNA approach, therapeutic procedures based on miRs embrace two different strategies including miR inhibition and miR replacement. The former strategy exploits antisense therapy to suppress the action of the endogenous miRs by using synthetic single-stranded RNAs acting as miR antagonists with sequences complementary to the endogenous miR [37]. The miR antagonists include anti-miRs, locked-nucleic acids (LNA) or antagomiRs carrying chemical modifications that magnify the affinity for the target miR and trap the endogenous miR in a configuration that cannot be incorporated into RISC complex, or cause degradation of the endogenous miR [38].

In the case of replacement strategy, synthetic miRs (miR mimics) are applied to mimic the function of the endogenous miRs causing mRNA inhibition/degradation, and exert a gene silencing effect [38].

Although siRNAs have been frequently used for silencing the target genes, and even a few siRNAs are studied in clinical trials [39], there are several fundamental disadvantages which considerably limit therapeutic use of siRNA methodology. The most important drawbacks include scrambling in the biogenesis of the endogenous miR, evocation of the interferon response, and the off-target effects [40–43]. On the other hand, the unique biogenesis and mechanism of miR action enables it to be freed from aforementioned limitations. Impressive advantages of miR-based tools, such as specificity and safety, and the fascinating feature of multiple-targeting potential nominate miRs as a useful therapeutic approach [44].

## miRs as Therapeutic Target and Tool for FH Therapy

Since miRs are known to have a pivotal role in LDL-C metabolism [45] and dysregulated miRs have been frequently found to be involved in the pathogenesis of FH and various CVDs, they have been suggested as potential targets for therapeutic intervention [46–49].

**Fig. 1:**
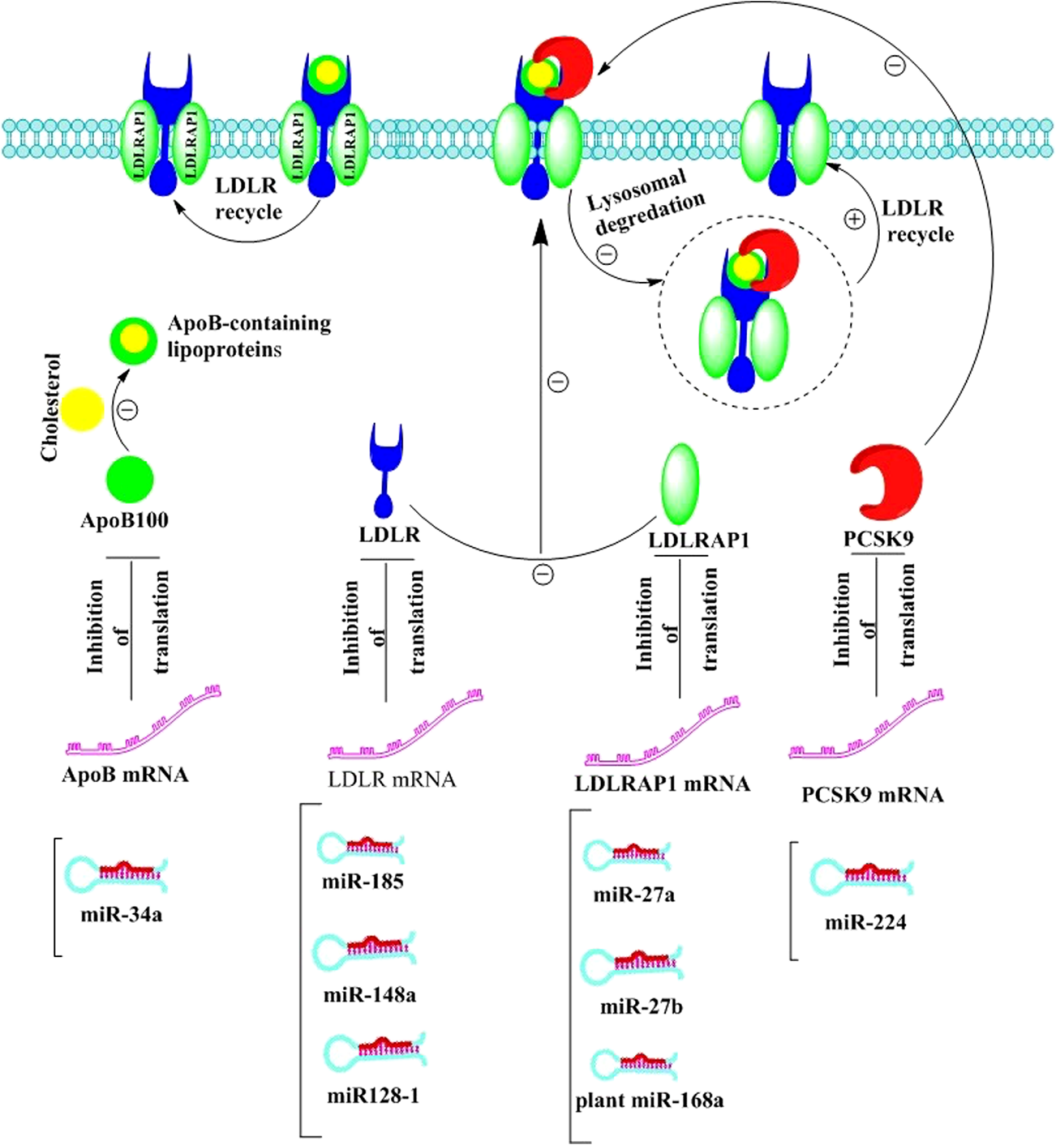
A schematic view of miR-mediated regulation of FH-related genes

Different lines of evidence support the legitimacy of considering miRs as therapeutic targets for FH teatmenttr. First, miR-based therapy with miR-34- and miR-122-based drugs has reached phase 2 clinical trial development [50–52]. Second, a number of in vitro and in vivo studies have revealed the importance of miRs in controlling plasma LDL-C through modulating LDLR, apoB, and PCSK9 expression. Hence, in attention to critical and well-established role of *LDLR*, *APOB*, and *PCSK9* genes in LDL-C hemostasis and metabolism, therapeutic manipulation of related miRs, via miR mimics or inhibitors, can be an efficient and attractive approach for lowering elevated LDL-C and reducing risk of CV events in FH patients (Fig. 1).

### LDLR-Targeting miRs

To date, a large number of mutations in the *LDLR* gene have been found to be associated with FH [5]. *LDLR* gene mutations may affect function and structure of LDLR protein leading to decreased affinity of the receptor to ligands such as apoB-containing lipoproteins [53] and/or to deficiency of LDLR internalization [54] which consequently result in abnormal accumulation of LDL-C in plasma [38].

As emerged bioinformatically by genome-wide association studies (GWAS), LDLR was predicted as a target gene for miR-130b, miR-301b, miR-148a, and miR-128-1, thereby they may have the potential to regulate circulating LDL-C levels [55]. In vitro studies on human and mouse hepatoma cells have shown that a large number of miRs, including miR-27a, miR-27b, miR-185, miR-148a, miR-128-1, miR-130b, and miR-301b, can directly modulate LDLR expression through post-transcriptional regulation via targeting the 3’ UTR of LDLR [56–60] (Table 1). Among these miRs, as revealed by in vivo studies on genetically modified mice, only miR128-1, miR-148a, and miR-185 could significantly change plasma LDL-C levels. Inhibition of miR128-1 and miR-148a expression markedly reduced circulating levels of plasma LDL-C in ApoE^−/−^ and Ldlr^−/+^ mice, respectively [58, 59]. Inhibition of miR-185 expression was also associated with significantly less plasma LDL-C levels and slow atherosclerotic plaque progression [60]. In addition to the aforementioned validated miRs, a wide range of miRs, including hsa-miR-130a-3p, bta-miR-17-5p, bta-miR-146a, bta-miR-146b, hsa-miR-27a-3p, hsa-miR-17-5p, and many other miRs have been predicted by Miranda to interact with the 3’ UTR region of LDLR mRNA. Finally, a recent study by He et al. revealed that miR-195 inhibited lipopolysaccharide-mediated intracellular cholesterol accumulation and LDL cell uptake by decreasing *LDLR* gene expression [62]. These findings illustrate that inhibition of miR128-1, miR-148a, and miR-185 might be an efficient therapy to decrease plasma LDL-C levels in patients with FH.

**Table 1:** An overview of putative therapeutic miRs validated to directly target FH-related pathogenic genes

Regulating miR	Validation method	Condition	Prediction program	Ref.
LDLR-regulating miRs				
miR-27a	Luciferase reporter assay, real-time PCR, Western blot	HepG2 cells	TargetScan, miRanda	[56]
miR-27b	Luciferase reporter assay, real-time PCR, Western blot	Cos-7 cells	TargetScan, miRWalk, miRanda	[57]
miR-128-1	Luciferase reporter assay, real-time PCR	HEK293T cells, C57BL/6 J and *Apoe* ^*−/−*^ mice	TargetScan	[58]
miR-130b				
miR-301b				
miR-148a	Luciferase reporter assay, real-time PCR, Western blot, ribonucleoprotein immunoprecipitation	HepG2 cells, Hepa cells, Huh7 cells	TargetScan, miRWalk, and miRanda	[59]
miR-185	Luciferase reporter assay, real-time PCR, Western blot	HepG2 cells	miRanda	[60]
PCSK9-regulating miRs				
miR-224	Luciferase reporter assay, real-time PCR, Western blot	U251 and U87 cells	DIANA microT	[61]
mi-27a	Luciferase reporter assay, real-time PCR, Western blot	HepG2 cells	TargetScan, miRanda	[56]
miR-195	overexpressing or knocking down miR-195, real-time PCR, Western blo	HepG2 and Huh7	–	[62]
miR-132	Luciferase reporter assay, real-time PCR, Western blot	LNCaP and C4-2B cells	TargetScan	[63]
miR-499	Luciferase reporter assay, real-time PCR, Western blot	HepG2 and Huh7 cells	miRBase	[64]
MiR-342	Luciferase reporter assay, real-time PCR, Western blot	LNCaP and C4-2B cells	TargetScan	[65]
miR-185	Luciferase reporter assay, real-time PCR, Western blot	HepG2 and THLE-2 cells	miRStart	[65, 66]
apoB-containing lipoproteins-regulating miRs				
miR-34a	Luciferase reporter assay	C57BL/6 mice	ND	[67]
miR-30c	Luciferase reporter assay	Cos-7 cells	TargetScan	[68]
	Luciferase reporter assay	Male C57Bl/6 J and *L-MTP* ^*-/-*^ mice, Huh7 cells	TargetScan	[69]
miR-122	Luciferase reporter assay	Hepa cells Gene knockout mice	TargetScan	[70]
	Luciferase reporter assay	AML 12 cells, HeLa cells	TargetScan	[71]
LDLRAP1- regulating miRs				
miR-27a	Luciferase reporter assay, real-time PCR, Western blot	HepG2 cells	TargetScan, miRanda	[56]
miR-27b	Luciferase reporter assay, real-time PCR, Western blot	Cos-7 cells	Targetscan, miRWalk, miRanda	[57]
miR168a	Luciferase reporter assay, real-time PCR, Western blot	C57BL/6 J mice, HepG2, and Caco-2 cells	ND	[72]

*ND* not define

### PCSK9-Targeting miRs

PCSK9 is an intriguing protein with a ubiquitous expression and a range of partially unexplored functions [73–75]. PCSK9 is well-known for its homeostatic role in cholesterol metabolism through regulation of LDLR fate via binding to the extracellular EGF-A domain of the hepatic LDLR and promoting its lysosomal degradation. In rare (less than 0.5%) of FH patients, a missense gain-of-function mutation in the *PCSK9* gene results in elevated plasma PCSK9 levels, increased LDLR degradation and impaired recycling leading to reduced LDLR-mediated clearance of LDL-C from the bloodstream and increased plasma LDL-C levels [76, 77].

PCSK9 inhibition has emerged as an effective, approved and marketed LDL-C-lowering therapy where monoclonal antibodies (mAbs) to PCSK9 profoundly reduce LDL-C in FH patients [78–82]{Sahebkar, 2013 #48; Sahebkar, 2013 #49}. Evolocumab (Repatha®) and alirocumab (Praluent®) are now approved by regulatory authorities in the USA, Europe, Japan, and many other countries for the treatment of HeFH where LDL-C is inadequately reduced by maximal doses of statins. In addition, evolocumab is approved at a dose of 420 mg monthly for the treatment of HoFH. Preliminary findings from post-hoc or exploratory analysis of phase III trials with evolocumab and alirocumab provided encouraging evidence that the LDL-C reductions resulted in further reductions in CVD when added to existing statin with or without ezetimibe therapy [83, 84]. The CVD benefit has recently been confirmed in the Further Cardiovascular Outcomes Research With PCSK9 Inhibition in Subjects With Elevated Risk (FOURIER) trial with evolocumab with 27,500 patients (15% risk reduction of primary endpoint and 20% significant risk reduction of key secondary outcomes) [85].

PCSK9 mAbs at the currently approved doses (140 mg biweekly or 420 mg monthly) have a relatively short duration of effect, necessitating every 2- or 4-week administration; however, this may not be a concern for the management of patients owing to the satisfactory adherence of patient to these mAbs in phase III trials [86–88]. In addition, the cost-effectiveness of PCSK9 mAbs for the treatment of FH, which could be life-long, is debatable [89]. These issues highlight the need for durable and less expensive PCSK9 inhibitors that could potentially serve as alternatives to PCSK9 mAbs in order to reduce the cost of treatment. miR-based therapeutic approaches can be regarded as such an alternative for PCSK9 inhibition.

Although there are not many verified miRs for *PCSK9* regulation, some recent studies have shown that PCSK9 can be regulated directly and indirectly by some miRs. As verified by luciferase reporter method, miR-224 directly targets PCSK9 mRNA and significantly downregulates its expression [61]. Notably, miR-27a was reported to target promoter region of the *PCSK9* gene and up-regulate its expression [56]. Also, miR-195 was able to increase *PCSK9* gene expression in liver cancer cells stimulated with lipopolysaccharide [62]. Such inducing effect of the miR is supported by evidence showing miRs can enhance expression of target genes via interaction with promoter region instead of 3’ UTR of the mRNAs [90] (Table 1).

Furthermore, *PCSK9* expression has been reported to be modulated by miRs regulating SREBP transcription factors. The proximal promoter of *PCSK9* gene harbors a functional sterol regulatory element (SRE) that is targeted by sterol-responsive element binding proteins (SREBPs) in response to alterations in intracellular cholesterol levels [76, 77]. miR-132 and miR-499 have been recently identified to indirectly downregulate SREBP-1c [63] that is known to be a PCSK9 transcription factor [64, 76]. It was also found that miR-185 and miR-342 can inhibit the expression of SREBP-1 and -2, and therefore, reduce cholesterol synthesis [65, 66]. Given that SREBPs are the crucial transcription factors upregulating *PCSK9* gene, miRs inhibiting these transcription factors can be potentially exploited for FH miR therapy using miR mimetics.

On the other hand, several miRs were recently predicted to bind to the 3′ UTR of *PCSK9* mRNA*.* These include miR-18a-5p, miR-148, miR-323-5P, miR-570, miR-584t, miR-663-b, miR-922, miR-3919, miR-3974, miR-4509, miR-4690-5p, miR-4732-5p, miR-4795-5P, miR-5586-3P, and miR-6134 [91]. There are also several miRs presented by miRTarBase database, which are predicted, by Miranda, to interact with 3′ UTR of PCSK9, including hsa-miR-335-5p, hsa-miR-124-3p, hsa-miR-215-5p, hsa-miR-192-5p, hsa-miR-6864-5p, hsa-miR-6515-5p, hsa-miR-4656, hsa-miR-6797-5p, hsa-miR-1249-5p, hsa-miR-6875-5p, hsa-miR-4721, hsa-miR-6761-5p, hsa-miR-3126-5p, hsa-miR-4802-5p, hsa-miR-4446-3p, hsa-miR-6873-5p, hsa-miR-7845-5p, hsa-miR-6856-5p, hsa-miR-6758-5p, hsa-miR-6882-5p, hsa-miR-1303, hsa-miR-744-3p, hsa-miR-6501-5p, hsa-miR-4423-5p, hsa-miR-367-5p, hsa-miR-6764-5p, hsa-miR-1304-3p, hsa-miR-1915-3p, hsa-miR-1208, and hsa-miR-193a-5p. The aforementioned miRs merit further studies to be experimentally verified as direct regulators of PCSK9.

### apoB-Containing Lipoproteins-Targeting miRs

LDL-C-lowering approaches acting via modulation of LDLR activity or expression are ineffective in patients with HoFH with LDLR activity less than 2% [92–94]. In this condition, elevated plasma cholesterol can be modulated via reducing production of atherogenic apoB-containing lipoproteins including LDL-C and lipoprotein(a). This approach has been found to lower plasma cholesterol in HoFH patients by reducing apoB expression using mipomersen, an antisense oligonucleotide (ASO), and by inhibiting the activity of MTP by using lomitapide [95–98]. Side effects of both mipomersen and lomitapide, such as fatty liver and elevation in plasma transaminases [99], call for the necessity of alternative agents—with different mechanisms of action—modulating plasma cholesterol without causing side effects.

Hepatic production of atherogenic apoB-containing lipoproteins has been identified to be regulated by three miRs, including miR-34a [67], miR-30c [68, 69], and miR-122 [70, 71] (Table 1). The miRs have been reported to reduce production of liver apoB-containing lipoproteins through inhibition of HNF4α and MTP mRNA expression. Both HNF4α and MPT have critical roles in the assembly of apoB-containing lipoproteins. HNF4α is a transcription factor that binds to the promoter regions of *APOB* and *MTP* genes to enhance their expression [100–102]. It was found that miR-34a reduces HNF4α levels causing reduction of apoB and MTP expression [67, 103]. MTP is an important chaperone that transfers lipids to apoB peptide [104, 105]. miR-30c inhibits MTP expression via binding to the 3′ UTR of MTP mRNA and induces post-transcriptional degradation leading to reduced assembly of apoB-containing lipoproteins [68, 69]. In the case of miR-122, it decreases MTP expression by yet an unknown mechanism [106].

In addition, a number of miRs proposed to interact with 3′ UTR of apoB100 mRNA has been predicted by Miranda and presented in miRTarBase database. These include mmu-miR-122-5p, hsa-miR-885-5p, hsa-miR-7151-5p, hsa-miR-6869-5p, hsa-miR-502-5p, hsa-miR-1911-5p, hsa-miR-499b-5p, hsa-miR-500b-3p, hsa-miR-6862-3p, hsa-miR-6784-3p, and hsa-miR-624-5p.

To sum up, overexpression of miR-122, miR-34a, and miR-30c, as well as other predicted miRs, can be therapeutically useful to reduce production and plasma levels of atherogenic apoB-containing lipoproteins independent of LDLR targeting in FH, particularly in homozygous patients. However, the interactions and effectiveness of these miRs needs to be experimentally verified by using miR mimetics.

### LDLRAP-Targeting miRs

Low-density lipoprotein receptor adaptor protein 1 (*LDLRAP1*) is a cytosolic protein containing an N-terminal phosphotyrosine-binding (PTD) domain that interacts with the cytoplasmic tail of the LDLR and facilitates circulating plasma LDL-C clearance. Through clathrin-mediated endocytosis of hepatic LDLR, PTD domain of LDLRAP1 binds to the internalization sequence of the cytoplasmic tail of LDLR, and acts as an adaptor for LDLR endocytosis in the hepatocytes. A rarely-occurring recessive form of FH, named autosomal recessive hypercholesterolaemia, is caused by loss-of-function mutations in *LDLRAP1* gene leading to defection in LDLR internalization and subsequently, the reduced clearance of plasma LDL-C [5, 107].

miR-27a, miR-27b, and plant miR-168a have been found to directly target *LDLRAP1* expression, and consequently modulate LDLR activity (Table 1). As validated by luciferase reporter assays, 3′ UTR of LDLRAP1 mRNA is directly and specifically targeted by miR-27a [56] and miR-27b [57]. Although in vitro study on human hepatic Huh7 cells revealed that over-expression of miR-27b can profoundly downregulate mRNA expression of *LDLRAP1* gene, in vivo study on wild type mice showed that modulation of miR-27b cannot affect plasma levels of LDL-C and other indices of lipid profile [57]. miR-27a was also found to downregulate mRNA level of LDLRAP1 in HepG2 cells [56], while its in vivo effect on plasma LDL-C is unknown and needs to be studied. Furthermore, miR168a is a plant miR abundant in rice and has been found to be one of the most enriched exogenous plant-based miRs in the sera of Chinese peoples. In vitro and in vivo mechanistical studies revealed that miR168a has a high degree of complementarity with the exon 4 of mammalian LDLRAP1, which can bind to the human or mouse LDLRAP1 mRNA, inhibit LDLRAP1 expression in hepatocytes, and result in reduced clearance of LDL-C from mouse plasma [72]. As predicted by Miranda algorithm, there are several miRs in miRTarBase database which are proposed to interact with 3′ UTR of LDLRAP1 mRNA, including hsa-miR-124-3p, mmu-miR-124-3p, hsa-miR-9-5p, hsa-miR-615-3p, and hsa-miR-92a-3p.

In summary, miR inhibition using antisense approach can be a useful strategy to suppress aforementioned miRs, resulting in enhanced LDLRAP1 activity and improved clearance of plasma circulating LDL-C.

## Concluding Remarks

miRs regulating critical genes closed to FH, including *LDL*, *APOB*, *PCSK9*, *and LDLRAP1*, can be considered as potential therapeutic targets for FH patients. LDLR can be upregulated through inhibition of miRs including miR128-1, miR-148a, and miR-185 and consequently enhances liver clearance of LDL-C. In the case of PCSK9, use of therapeutic miRs mimic miR-224, miR-132 and miR-499, miR-185, and miR-342, and also inhibition of miR-27a can downregulate PCSK9 expression, and therefore, can also lower elevated LDL-C in FH patients. Notably, reduction of atherogenic apoB-containing lipoproteins, as an efficient therapeutic approach in HoFH, can be achieved via miR-122, miR-34a and miR-30c mimics, which are known to reduce atherogenic lipoproteins independent of LDLR. In the case of LDLRAP1, inhibition of miR-27a and miR-27b can be a reliable and efficient approach for upregulation of LDLRAP1 protein and enhancing LDLR activity leading to improved clearance of LDL-C from sera of hypercholesterolemic patients. Overall, among the aforementioned miRs, miR-27a is suggested to be the most putative therapeutic target as it can regulate three of the 4 important photogenic genes in FH patients including *LDLR*, *PCSK9*, and *LDLRAP1*.
